# Early intrathecal dexamethasone and methotrexate as an effective approach for immune effector cell-associated neurotoxicity syndrome after CAR-T cell therapies

**DOI:** 10.3389/fimmu.2025.1716317

**Published:** 2025-12-17

**Authors:** Juanxia Meng, Hairong Lyu, Zhao Wang, Xue Bai, Haibo Zhu, Yedi Pu, Xiaoyuan He, Xia Xiao, Mingfeng Zhao

**Affiliations:** Department of Hematology, Tianjin First Central Hospital, Tianjin Thrombosis and Hemostasis Institute, Tianjin, China

**Keywords:** CAR-T, immune effector cell-associated neurotoxicity syndrome (ICANS), intrathecal dexamethasone and methotrexate, efficacy, safe

## Abstract

**Introduction:**

Chimeric antigen receptor T (CAR-T) cell therapies have demonstrated remarkable success in treating relapsed and refractory (R/R) B-cell hematological malignancies. Although the application of CAR-T therapy in acute myeloid leukemia (AML) is still restricted, we and other institutions have also demonstrated high complete remission rate of CAR-T targeting C-type lectin-like molecule 1 (CLL1) for R/R AML. However, CAR-T cell related toxicities such as steroid-refractory and severe immune effector cell-associated neurotoxicity syndrome (ICANS) can be life-threatening. Previous cases have reported potential efficacy of intrathecal corticosteroids alone or in combination with chemotherapy. Theoretically, intrathecal corticosteroids combined with intrathecal chemotherapy can control ICANS faster and better. Whether intrathecal dexamethasone and methotrexate (IDM) is beneficial for the patient with steroid-refractory or severe ICANS remains unclear.

**Methods:**

We retrospectively analyzed the clinical data of 13 patients with severe or steroid-refractory ICANS, and evaluated the effect of IDM in the treatment of severe ICANS or steroid-refractory ICANS by analyzing the changes in ICANS grade, ICE score, and laboratory indicators. Grade 3–4 ICANS downgraded to grade 1 and grade 1–2 ICANS recovery to an ICE score of 10 points is considered ICANS remission.

**Results:**

Among the 13 patients, there were 7 cases of AML, 3 cases of ALL, 1 case of MM, 1 case of B-cell lymphoma, and 1 case of blastic plasmacytoid dendritic cell neoplasm, with a median age of 39 (11-65) years. The median number of prior lines of therapy was 7(1-16). There were 6 CAR-T products targeting CD19, CLL1, CD7, CD123, BCMA, and CD19-CD22, respectively. The median CAR-T cell infusion dose was 2.0×10^6^/kg. The median bone marrow blasts before lymphocyte depletion was 33.23%(5.34%-78.50%) and 9 patients had CNS involvement. All patients developed grade 1–2 CRS. Of the 13 patients, 5 had grade 3–4 ICANS and 8 had grade 1–2 ICANS. The median onset time of ICANS after CAR-T was 11(3-20) days. The median time of first IDM after ICANS was within 12 hours, and the median number of IDM was 1(1-3). The median time to ICANS remission was 1(1-7) days, with a response rate of 92.3% (12/13 patients). IDM significantly reduced CAR-T cells and tended to reduce protein and IL-6 levels in cerebrospinal fluid. As of June 30, 2025, the median OS and median PFS were 6 months and 5 months, respectively.

**Conclusion:**

Our data suggest that early administration of IDM may contribute to a rapid resolution of severe or steroid-refractory ICANS after CAR-T cell therapies, which may create opportunities for subsequent treatments in these patients. Larger sample and multicenter clinical trials are warranted to further validate these findings.

## 1 Introduction

Chimeric antigen receptor T (CAR-T) cell therapies have dramatically altered the landscape of cancer treatment and have achieved remarkable success in treating relapsed and refractory (R/R) B-cell hematologic malignancies, while also showing therapeutic potential in solid tumors (1). Several CAR-T cell products targeting CD19 or B-cell maturation antigen (BCMA) have been approved by FDA for application in acute lymphoblastic leukemia (ALL), B cell lymphoma and multiple myeloma (MM) (2, 3), respectively. Although the application of CAR-T therapy in acute myeloid leukemia (AML) is still restricted, we and other institutions have also demonstrated high complete remission rate of CAR-T targeting C-type lectin-like molecule 1 (CLL1) for R/R AML (4–6).

Despite CAR-T cell therapies hold promise for R/R hematological malignancies, some toxicities related to CAR-T such as cytokine release syndrome (CRS) and immune effector cell-associated neurotoxicity syndrome (ICANS) can be troublesome for physicians, particularly severe ICANS which can be life-threatening to patients (7). ICANS typically manifests as delirium, encephalopathy, aphasia, lethargy, attention deficit, agitation, tremor, seizures and potentially lethal cerebral edema and even coma (8). The incidence of ICANS in published randomized control trials for the approved CAR-T products varies from 15% to 65% (9). A systematic review summarized the clinical characteristics of ICANS including 23 observational studies with 1666 participants suffered from B cell malignancies who received CAR-T therapy and reported the incidence of ICANS among 19 of the 23 studies ranged from 37.5% to 77% (10). Previous studies have reported that severe ICANS may occur in 12-30% of patients receiving CD19 CAR-T therapy (11), and in 12% of patients with MM receiving BCMA CAR-T cell infusion (12). However, limited data are available for ICANS due to CLL1-targeted CAR-T cell therapy. In our previous study, 32 patients with R/R AML received anti-CLL1 CAR-T therapy, and 25% of patients experienced ICANS, two of whom developed severe ICANS of grade 3-4 (6). Another study reported that 1 among 7 childhood patients receiving anti-CLL1 CAR-T therapy experienced ICANS of grade 2 with transient attention loss, speech impediment, and tremors (13). CAR-T cell therapies with other targets such as CD7 or dual-target CAR-T are also at risk of varying degrees of ICANS (14, 15).

At present, systemic corticosteroids especially dexamethasone is the first-line pharmacotherapy for ICANS according to the American Society for Transplantation and Cellular Therapy (ASCTC) guidelines (8). However, high cumulative doses of corticosteroids are associated with inferior progression-free survival (PFS) and overall survival (OS) in these patients (16). Previous studies have showed that patients with B-ALL or B cell lymphoma receiving CAR-T therapy targeting CD19 or CD22, and experiencing ICANS refractory to systemic steroids can gain favorable outcomes after intrathecal corticosteroids or chemotherapy (17–19). Ji et al. have reported that earlier initiation of intrathecal dexamethasone is the optimal management of ICANS resulting from CAR-T cell therapy (16). Whether patients suffered from steroid-refractory or ICANS can be beneficial from intrathecal dexamethasone and methotrexate (IDM) remains unclear. Here, we conducted a retrospective analysis in a series of patients who developed ICANS after receiving different CAR-T products to evaluate the efficacy and safety of IDM.

## 2 Materials and methods

### 2.1 Study design and patient information

The study is a retrospective analysis including a total of 13 patients with R/R malignant hematological tumors who underwent CAR-T cell treatment from January 2021 to December 2024 in Tianjin First Central Hospital. The inclusion criteria were patients who received CAR-T cell therapy with different targets, developed severe ICANS (grade 3-4) or steroid-refractory grade 1–2 ICANS. ICANS was graded based on the ASCTC consensus grading system. According to previous criteria, steroid-refractory ICANS was defined as any patient who did not show clinical signs of improvement after at least one dose of corticosteroids within at least 1 day (median 24 hours) (20).All patients were treated with IDM regimen, which included intrathecal of 10mg dexamethasone and 10mg methotrexate. This study was conducted according to the principles of the Declaration of Helsinki and with the approval of the Ethics Committee of Tianjin First Central Hospital. All the enrolled patients or their families provided written informed consent.

### 2.2 Evaluation of clinical response and outcomes

The clinical response status of ICANS was evaluated using the ASCTC consensus grading system and the Immune Effector Cell Encephalopathy(ICE)Score (21). Here, a decrease in ICANS from grade 3–4 to grade ≤ 1 and a decrease in ICANS from grade 1–2 to an ICE score of 10 points are considered ICANS remission. The median PFS and OS were calculated to assess the clinical outcomes. OS was defined as the time from the start of CAR-T cell infusion to death from any cause, while PFS was defined as the time from the start of CAR-T cell infusion to disease progression, death, or the last follow-up (whichever occurred first). The endpoint for follow-up was June 30th, 2025.

### 2.3 Serum cytokine analysis

Serum samples of cytokines including IL-2, IL-4, IL-6, IL-10, TNF-α and IFN-γ as well as inflammatory markers such as C-reactive protein (CRP), ferritin and lactate dehydrogenase (LDH) were frequently detected after CAR-T cell infusion. The concentrations of inflammatory markers in serum or cerebrospinal fluid were evaluated by Luminex assay, according to the manufacturer’s instructions.

### 2.4 Detection of IL-6, protein and CAR-T cells in cerebrospinal fluid

IL-6, protein, and CAR-T cells from the cerebrospinal fluid were collected and tested each time intrathecal injections were performed. IL-6 in cerebrospinal fluid was evaluated by Luminex assay, according to the manufacturer’s instructions. The level of protein in cerebrospinal fluid was detected by biochemical examination. The number of CAR-T cells in cerebrospinal fluid was assessed via flow cytometry.

### 2.5 Statistical analysis

Patient and disease characteristics were mostly summarized using descriptive statistics. Differences between two groups were tested using Student’s t-test or Mann-Whitney test for continuous variables depending on the distribution of the data, and χ2 test or Fisher exact test for categorical variables. The statistical analysis was conducted using GraphPad Prism V.9 (GraphPad Software, San Diego, California, USA). P *<* 0.05 was considered as statistically significant.

## 3 Results

### 3.1 Patient characteristics

A total of 13 patients with R/R malignant hematological tumors treated with CAR-T cells were included in this retrospective analysis, including 7 patients with AML, 3 cases of ALL, 1 case of MM, 1 case of B-cell lymphoma, and 1 case of blastic plasmacytoid dendritic cell neoplasm (**Table 1**). Of the 13 patients, 6 were treated with CD19 CAR-T, 3 with CLL1 CAR-T, and the other 4 received BCMA CAR-T, CD7 CAR-T, CD123 CAR-T, and CD19 CD22 CAR-T, respectively. The study included 7 female and 6 male participants, with a median age of 39 years and a range of 11–65 years. The median ECOG score of patients before CAR-T treatment was 2 points, ranging from 1–3 points. The median number of prior lines of therapy was 7, ranging from 4 to 16 lines, and 4 patients had received allogeneic hematopoietic stem cell transplantation (HSCT) before CAR-T therapy (**Table 1**). The median bone marrow blasts at baseline accounted for 33.23%, ranging from 5.34% to 78.50%, of which 9 patients had CNS leukemia. Prior to CAR-T cell infusion, all patients received fludarabine and cyclophosphamide regimen for lymphodepletion. The median CAR-T cell dose received by all patients was 2.0×10^6^/kg, with a range of 0.5-3.0×10^6^/kg (**Table 1**).

**Table 1:** Clinical characteristics of patients.

Characteristics	N=13
Age (years), median (range)	39 (11–65)
Gender, female/male	7/6
ECOG score (points), median (range)	2(1-3)
Disease type	
Acute myeloid leukemia	7
Acute lymphoblastic leukemia	3
Multiple myeloma	1
B cell lymphoma	1
Blastic plasmacytoid dendritic cell neoplasm	1
Prior lines of therapy, median (range)	7(4-16)
HSCT before CAR-T	4
CAR-T target	
CD19 CAR-T	6
CLL1 CAR-T	3
BCMA CAR-T	1
CD7 CAR-T	1
CD123 CAR-T	1
CD19+CD22+ CAR-T	1
CAR-T cell infusion dose (10^6^/kg), median (range)	2.00(0.50-3.00)
BM blasts before lymphocyte depletion (%), median (range)	33.23(5.34-78.50)
CNS involvement	9

ECOG, Eastern Cooperative Oncology Group; HSCT, hematopoietic stem cell transplantation; CAR-T, Chimeric Antigen Receptor T-Cell; BM, bone marrow; CNS, central nervous system; CRS, cytokine release syndrome; ICANS, immune effector cell-associated neurotoxicity syndrome.

### 3.2 Clinical response of ICANS

**Figure 1:**
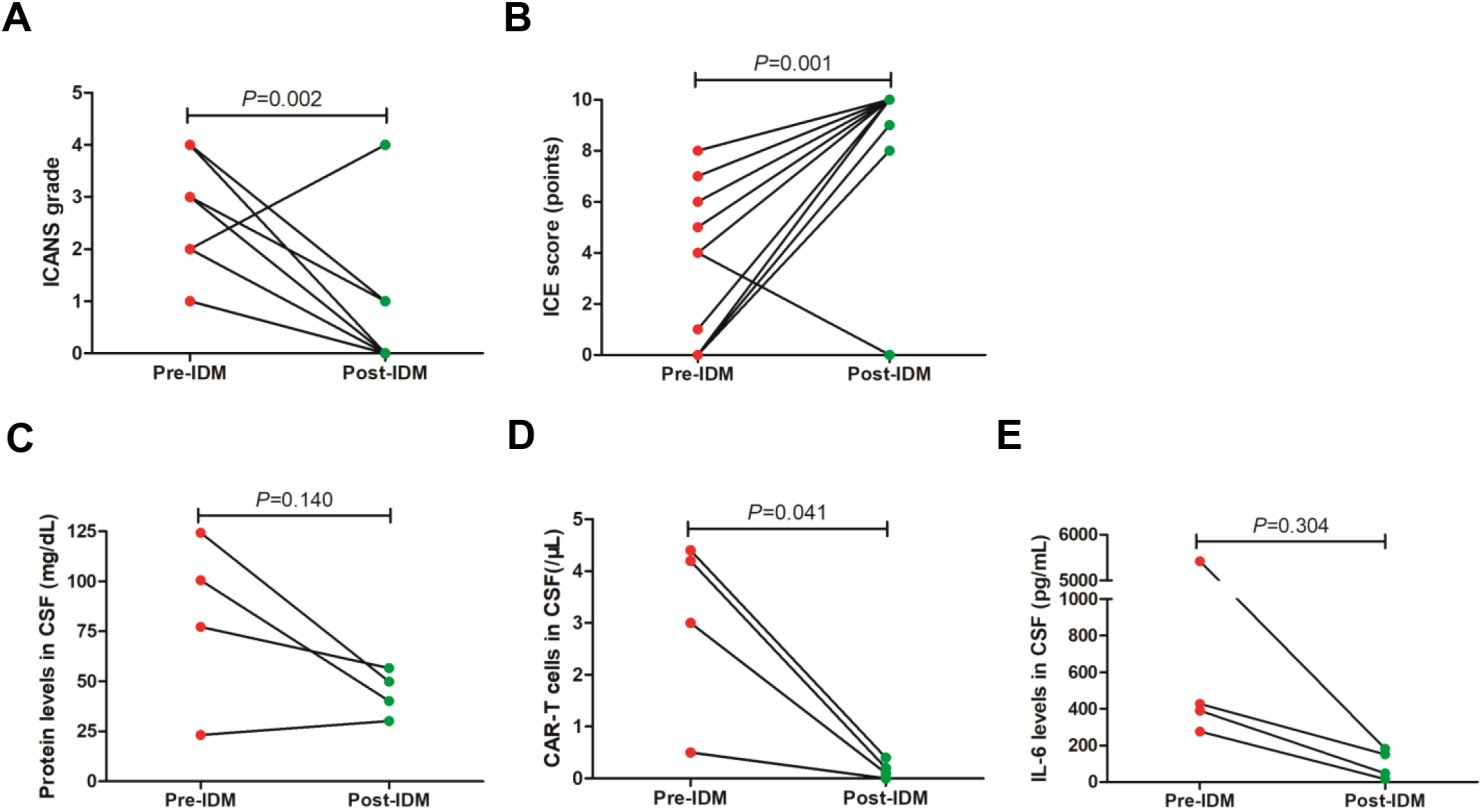
Clinical response of ICANS after IDM. **(A, B)** ICANS grade and ICE score improved significantly after IDM treatment. **(C-E)** Changes of cytokines, protein levels and CAR-T cells in CSF after IDM treatment. ICANS, immune effector cell-associated neurotoxicity syndrome; IDM, intrathecal dexamethasone and methotrexate; ICE, immune effector cell-associated encephalopathy; CSF, cerebrospinal fluid; CAR-T, chimeric antigen receptor T cell.

All patients had CRS reactions after CAR-T cell infusion, including 2 cases with grade 1 CRS and 11 patients with grade 2 CRS, followed by varying degrees of ICANS (**Table 2**). The median onset time of ICANS post CAR-T infusion was 11 days, ranging from 3 days to 20 days (**Table 2**). Of the 13 patients, 5 had grade 3–4 ICANS and 8 had grade 1–2 ICANS, the latter of which was refractory to intravenous dexamethasone. The median duration of intravenous dexamethasone in patient with grade 1–2 ICANS before first IDM was 4.5 days with a range of 3–7 days. For patients with severe ICANS, they received the first IDM in combination with intravenous dexamethasone within 24 hours, and four of them received their first IDM within 12 hours. For patients with steroid-refractory grade 1–2 ICANS, the median time to first IDM was within 24 hours, and the first IDM time was no more than 72 hours in all patients. Among the 13 patients, 12 achieved ICANS remission (**Table 2**). As shown in **Figures 1A, B**, patients in remission had significant improvement in ICANS grade and ICE score after IDM treatment. The median IDM number was 1, ranging from 1 to 3. Four patients received at least one dose of IDM treatment, and three of them were patients with severe ICANS. Among patients in remission, all patients achieved rapid response, with a median response time of 1 day after the first IDM, ranging from 1 to 7 days (**Table 2**).

**Table 2:** The efficacy of IDM in the management of ICANS.

Characteristics	N=13
CRS grade	
Grade 1	2
Grade 2	11
ICANS grade	
Grade 1	3
Grade 2	5
Grade 3	3
Grade 4	2
Onset time of ICANS after CAR-T (days), median (range)	11(3-20)
Duration of intravenous dexamethasone before first IDM (days), median (range)	6(1-7)
Time to first IDM after ICANS	
Within 12 hours	7
Within 12–24 hours	3
Within 24–48 hours	2
Within 48–72 hours	1
Number of ICANS remission	12
Number of IDM, median(range)	1(1-3)
Time to grade 1 ICANS or ICE score of 10 points after IDM (days), median(range)	1(1-7)

IDM, intrathecal dexamethasone and methotrexate; ICANS, immune effector cell-associated neurotoxicity syndrome; ICE, immune effector cell-associated encephalopathy.

### 3.3 Changes of cytokines, protein levels and CAR-T cells after IDM

**Figure 2:**
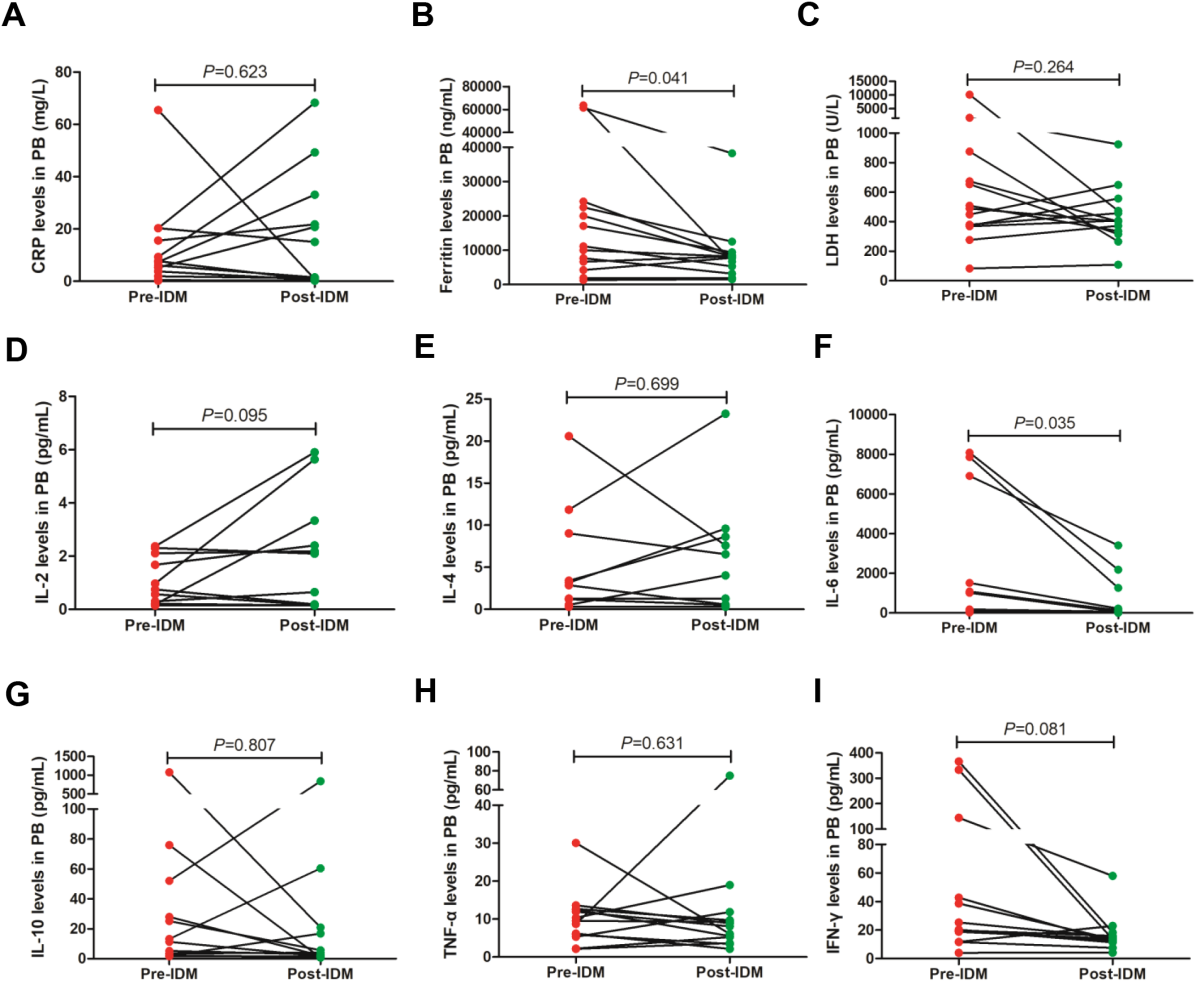
Changes of cytokines and inflammatory markers in PB pre- and post-IDM. Graphs **A-I** display CRP, Ferritin, LDH, IL-2, IL-4, IL-6, IL-10, TNF-a, and IFN-g levels, respectively. CRP, C-reactive protein; LDH, lactate dehydrogenase; PB, peripheral blood; IDM, intrathecal dexamethasone and methotrexate.

To assess the possible toxicity related to IDM, serum specimens before and after IDM were collected to analyze changes in cytokines and inflammatory markers, such as IL-6, CRP, LDH and ferritin. For the 4 patients received more than one dose of IDM treatment, we also collected cerebrospinal fluid specimens to evaluate changes of IL-6, protein levels and CAR-T cells related to IDM. We found that protein levels in cerebrospinal fluid showed a downward trend after IDM, although there was no statistical difference (**Figure 1C**). Similarly, IL-6 levels in cerebrospinal fluid can be observed a downward trend post IDM (**Figure 1E**). However, IDM treatment can significantly reduce CAR-T cells in cerebrospinal fluid (**Figure 1D**). And there may be a statistical significance about IL-6 and protein levels in cerebrospinal fluid by expanding the sample size. Next, we analyzed inflammatory factors in the blood and found that IDM treatment significantly reduced serum ferritin levels (**Figure 2B**). We also found a significant decrease in serum IL-6 levels after IDM treatment (**Figure 2F**). However, other cytokines and inflammatory markers, including IL-2, IL-4, IL-10, TNF-α, IFN-γ, CRP and LDH did not change significantly prior and post IDM (**Figure 2**). In summary, IDM can rapidly improve severe or steroid-refractory ICANS, and can reduce levels of IL-6 and ferritin in peripheral blood, as well as CAR-T cell, protein, and IL-6 levels in cerebrospinal fluid. IDM was well tolerated by all patients and no significant adverse effects were observed. Therefore, IDM can be used as a safe and effective way to control ICANS.

### 3.4 Clinical outcomes

**Figure 3:**
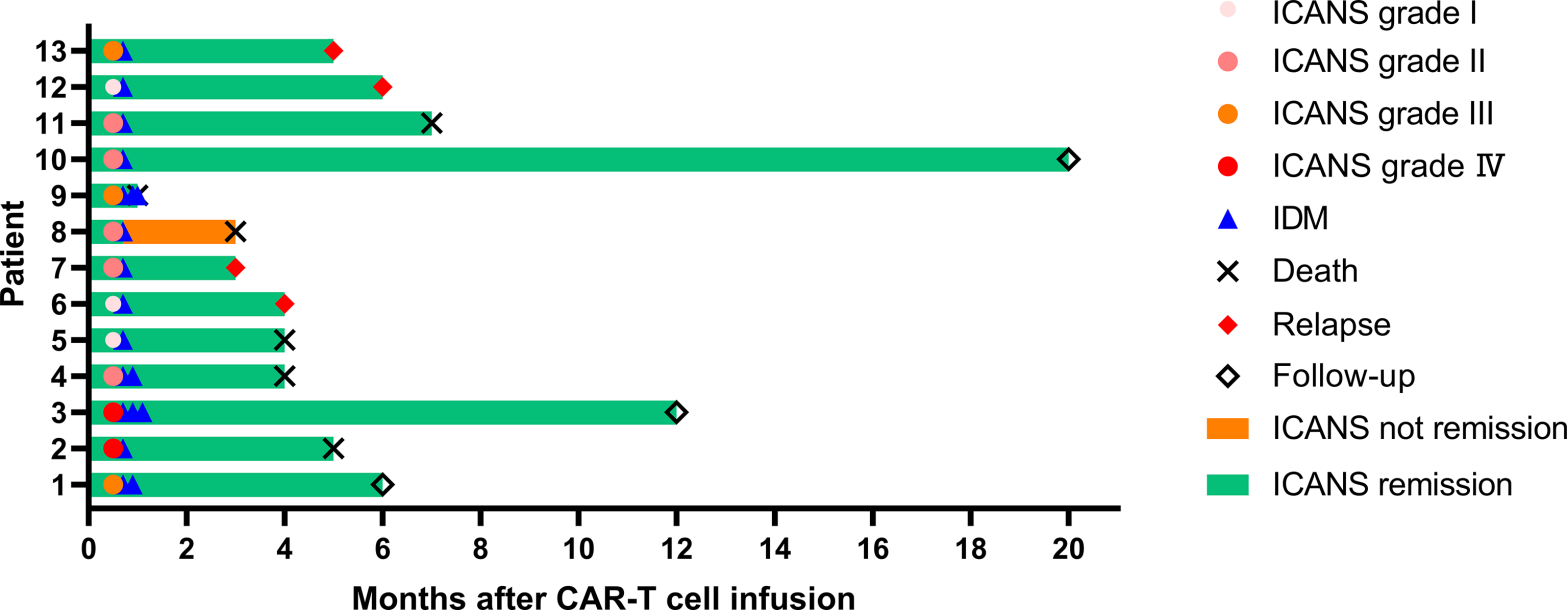
Clinical outcomes of all patients. ICANS, immune effector cell-associated neurotoxicity syndrome; IDM, intrathecal dexamethasone and methotrexate.

In this retrospective study, 13 patients were collected to evaluate the impact of IDM on severe or steroid-refractory ICANS, of which 12 patients achieved ICANS remission (**Figure 3**). The patient who didn’t achieve remission from ICANS had severe infection before IDM and eventually died of septic shock. All 12 patients achieved bone marrow remission at 1 month, and ICANS was also in remission. Until June 30, 2025, there were 3 patients in disease-free at 6 months, 12 months and 20 months of follow-up, respectively. Three patients died of severe infection at 4 months, 4 months, and 7 months after CAR-T cell therapy, respectively. One patient died of respiratory failure 5 months after CAR-T cell infusion. And one AML patient was bridged to allogeneic hematopoietic stem cell transplantation after receiving CLL-1 CAR-T cell infusion and died of serious transplant-related complications 1 month after CAR-T therapy. Another 4 patients underwent disease recurrence within 6 months post CAR-T treatment. As of June 30, 2025, the median OS and PFS of these patients were 6 months and 5 months, respectively.

## 4 Discussions

CAR-T cell therapies have demonstrated impressive efficacy in patients with R/R hematologic malignancies (1, 4), showing great potential for treating solid tumors (22–24), as well as refractory autoimmune conditions such as autoimmune hemolytic anemia and systemic lupus erythematosus (25–27). However, CAR-T related toxicities including severe CRS and ICANS can be fatal. Therefore, it is important to differentiate severe CRS and ICANS from infection, central nervous system disease or other factors. Generally, ICANS manifests from minor headaches, confusion, handwriting changes to aphasia, seizures, cerebral edema, and even coma requiring mechanical ventilation (28). ICANS usually occurs within 1 to 3 weeks after CAR-T cell therapy, which is similar to the onset time of ICANS in this study, while delayed ICANS can also be observed. ICANS was previously considered part of the spectrum of CRS. However, ICANS is now diagnosed, graded, and managed as a distinct pathophysiologic process from CRS due to its more delayed onset and its refractoriness to IL-6 receptor antibody.

The first-line therapy of CRS still depends on corticosteroids and tocilizumab, while steroids remain the mainstay of treatment for ICANS. Most cases of grade 1–2 ICANS are reversible and can be resolved with supportive care, but severe ICANS can be life-threatening. To prevent severe CRS or ICANS, pre-emptive tocilizumab and/or steroids were applied without much negative impact on the efficacy of CAR-T therapy although the possible suppression of CAR-T cell activity (29, 30). According to the study by Tim Lakomy et al. (31), early use of corticosteroids plus tocilizumab for low-grade CRS after CD19 CAR-T therapy may significantly reduce progression from low- to high-grade CRS, without worsening the incidence and severity of ICANS or the outcome of CAR-T therapy.

At present, high dose of intravenous dexamethasone is the major approach for severe ICANS, which can lead to adverse effects especially the high risk of serious infection. Furthermore, ICANS refractory to systemic steroids can be observed after CAR-T therapy. Therefore, it is urgently required to identify safe and effective second-line measures to manage severe ICANS or steroid-refractory ICANS. Although anakinra, an IL-1R antagonist, combined with corticosteroid and/or tocilizumab could improve clinical and inflammatory indices in steroid-refractory ICANS without or with CRS after CD19 CAR-T therapy, the improvement of neurotoxicity seemed limited (20). Another study showed that high dose of anakinra at 12mg/kg/day could mitigate grade 2 or higher ICANS whereas the resolution from the time of anakinra initiation was 7 days (32). Different with tocilizumab, siltuximab as a small molecule that can directly bind circulating IL-6 and cross blood-brain barrier may be a prior recommendation to control refractory ICANS (33). In a case report, siltuximab alone successfully managed a grade 2 ICANS patient who received talquetamab, a T cell redirecting bispecific antibody that targets G protein-coupled receptor, class C group 5 member D (GPRC5D) on MM cells and CD3 on T cells (34). Moreover, Pan J et al. attempted to manage steroid-refractory CRS by ruxolitinib for its ability to down regulate inflammatory cytokines including IL-1, IL-6, TNF-α and IFN-γ and demonstrated its effectiveness and safety in CRS (35). In this study, patient 2 and patient 13 experienced grade 4 and grade 3 ICANS, respectively. They both received ruxolitinib and intravenous dexamethasone before undergoing IDM treatment, but their clinical symptoms did not improve. However, after receiving IDM treatment, their neurotoxicity symptoms were rapidly relieved. Due to the small sample size, the efficacy of ruxolitinib in severe or steroid-refractory ICANS still requires further investigation.

However, there are currently no guidelines for the treatment of steroid-refractory ICANS. Since CAR-T cells can enter the central nervous system and cause an inflammatory response, intrathecal corticosteroids appear to be a feasible treatment for ICANS. Indeed, Shalabi et al. have reported that 7 of 79 patients with relapsed/refractory B-ALL who received CAR-T therapy targeting CD19 or CD19/CD22 experienced severe ICANS, among whom six received systemic steroids as first-line therapy. Subsequently, 5 out of 6 were treated with intrathecal hydrocortisone due to rapid progression of neurological symptoms and gained rapidly resolution from severe ICANS within 24 hours (18). Similar to our findings, IDM can quickly alleviate ICANS with a median response time of 1 day. Another article reported that two patients with DLBCL experiencing grade 4 ICANS refractory to high dose of systemic steroids after CD19 CAR-T cells therapy, got rapid neurological recovery after treatment with intrathecal hydrocortisone (17), demonstrating the effectiveness of intrathecal hydrocortisone for severe ICANS. Ji and colleagues reported earlier intrathecal dexamethasone can also significantly shorten the recovery time of ICANS without affecting the efficacy of CAR-T cell therapy (16). One case series concluded that among patients with high-grade or steroid-refractory ICANS, patients who received early intrathecal steroids had higher PFS and OS than those who did not or received late intrathecal therapy (36).

Methotrexate can inhibit the proliferation of activated lymphocytes and alleviate the function of CD8^+^ T cells. Theoretically, intrathecal corticosteroids in combination with intrathecal methotrexate can control ICANS faster and better. However, limited data are available for IDM in the management of severe or steroid-refractory ICANS. Here, we retrospectively analyzed 13 cases of severe or steroid-refractory ICANS and found that IDM could rapidly resolve ICANS without uncontrollable adverse effects, with a median response time of 1 day and a response rate of 92.3%. ICANS is known to be attributed to blood-brain barrier disruption due to elevated levels of inflammatory cytokines and immune effector cells in cerebrospinal fluid. We also found that IDM significantly reduced CAR-T cells and tended to reduce protein and IL-6 levels in cerebrospinal fluid, which may further explain the role of IDM in the treatment of severe or steroid-refractory ICANS. Ji and colleagues conducted a study on the patients with ICANS (≥1) after CAR-T cell therapy who were assigned to either the intrathecal (IT) dexamethasone group or the non-IT group (16). They observed that inflammatory cytokines and biomarkers decreased significantly and rapidly after 24 h of IT dexamethasone treatment, and 83.3% (15/18) of patients recovered from neurotoxicity. Compared with Ji’s study, the remission rate of IDM in this study seems to be higher than that of IT dexamethasone. However, due to our small sample size and lack of a control group, more participants and randomized controlled trials are needed to validate these results. Although the targets of CAR-T vary, IDM has the potential to control ICANS following various CAR-T therapies, suggesting that this treatment may have broad applicability. In theory, a stratified analysis of the efficacy of different CAR-T products would yield more convincing results. However, due to the small sample size, the present study is not suitable for stratified analysis. We will expand the sample size, extend the follow-up period, and perform stratified analysis in the future.

Altogether, this study suggests that early administration of IDM may contribute to a rapid resolution of severe or steroid-refractory ICANS after CAR-T cell therapies, which may create opportunities for subsequent treatments in these patients. Given the lack of first-line recommendation for severe ICANS or steroid-refractory ICANS, and its poor prognosis, more strategies are urgently warranted. Large samples and prospective studies are needed to validate the efficacy and safety of IDM in the treatment of severe or steroid-refractory ICANS.

## Data Availability

The original contributions presented in the study are included in the article/supplementary material. Further inquiries can be directed to the corresponding authors.
